# Photodynamic technology for delineating surgical margins in recurrent extramammary Paget’s disease: a case report

**DOI:** 10.3389/fmed.2026.1850030

**Published:** 2026-05-08

**Authors:** Xuan Guo, Haixia Wang, Xueqing Wang, Nan Cao, Zhiqiang Zhang, Shengli Chen, Guangliang Zhang

**Affiliations:** 1 Dermatology Hospital of Shandong First Medical University, Jinan, Shandong, China; 2 Shandong Provincial Institute of Dermatology and Venereology, Shandong Academy of Medical Sciences, Jinan, Shandong, China

**Keywords:** cutaneous tumor, dermatologic surgery, extramammary Paget’s disease, Mohs, photodynamic diagnosis (PDD)

## Abstract

**Background:**

Extramammary Paget’s disease (EMPD) is a rare cutaneous malignancy characterized by a high propensity for recurrence and metastasis. EMPD typically presents as slowly progressive, ill-defined erythematous plaques, which are often misdiagnosed as inflammatory dermatoses, leading to delayed diagnosis. Surgical excision remains the standard treatment for EMPD; however, due to ambiguous lesion borders, positive surgical margins frequently occur, resulting in a high local recurrence rate. In this study, we aimed to evaluate the clinical efficacy and safety of photodynamic diagnosis (PDD) in assisting surgical margin delineation for cutaneous tumors.

**Methods:**

A retrospective analysis was conducted on a patient with histologically confirmed scrotal EMPD. Despite multiple prior Mohs procedures and wide excisions, the disease relapsed. Ultimately, fluorescence-guided photodynamic technology was adopted to precisely demarcate tumor boundaries, followed by surgical excision according to the delineated margins.

**Results:**

Postoperative histopathological examination confirmed clear surgical margins and complete tumor resection. The patient is under continuous follow-up.

**Conclusion:**

Photodynamic technology facilitates accurate tumor margin delineation, guidessurgical resection extent, reduces recurrence risk, and improves patient comfort.

## Introduction

Extramammary Paget’s disease (EMPD) is a rare cutaneous malignancy. Its incidence in China is 0.4 per 100,000 individuals. It mainly occurs in the perianal and genital regions of elderly patients. The clinical manifestations are mostly pink or erythematous patches, which are easily misdiagnosed as benign conditions such as eczema, psoriasis, and contact dermatitis. Patients often ignore it, and clinicians are also prone to misdiagnosis, resulting in diagnostic delays of several years (1–3).

Surgical resection is currently the preferred treatment method for EMPD. However, due to the multifocal nature, irregular morphology, and poorly defined boundaries of tumors, the rate of positive surgical margins is relatively high, and local recurrence is common. Although Mohs microscopic mapping surgery is recommended to control the margins, due to the irregular infiltration pattern of the tumor, its recurrence rate is still higher than that of other skin malignancies. Therefore, exploring more precise preoperative margin positioning methods has significant clinical significance (1, 4, 5). Herein, we report a case of recurrent extramammary Paget’s disease treated with Mohs surgery combined with photodynamic technology. This approach reduced the number of operations, shortened hospital stays, and achieved satisfactory wound healing outcomes.

## Case report

**Figure 1:**
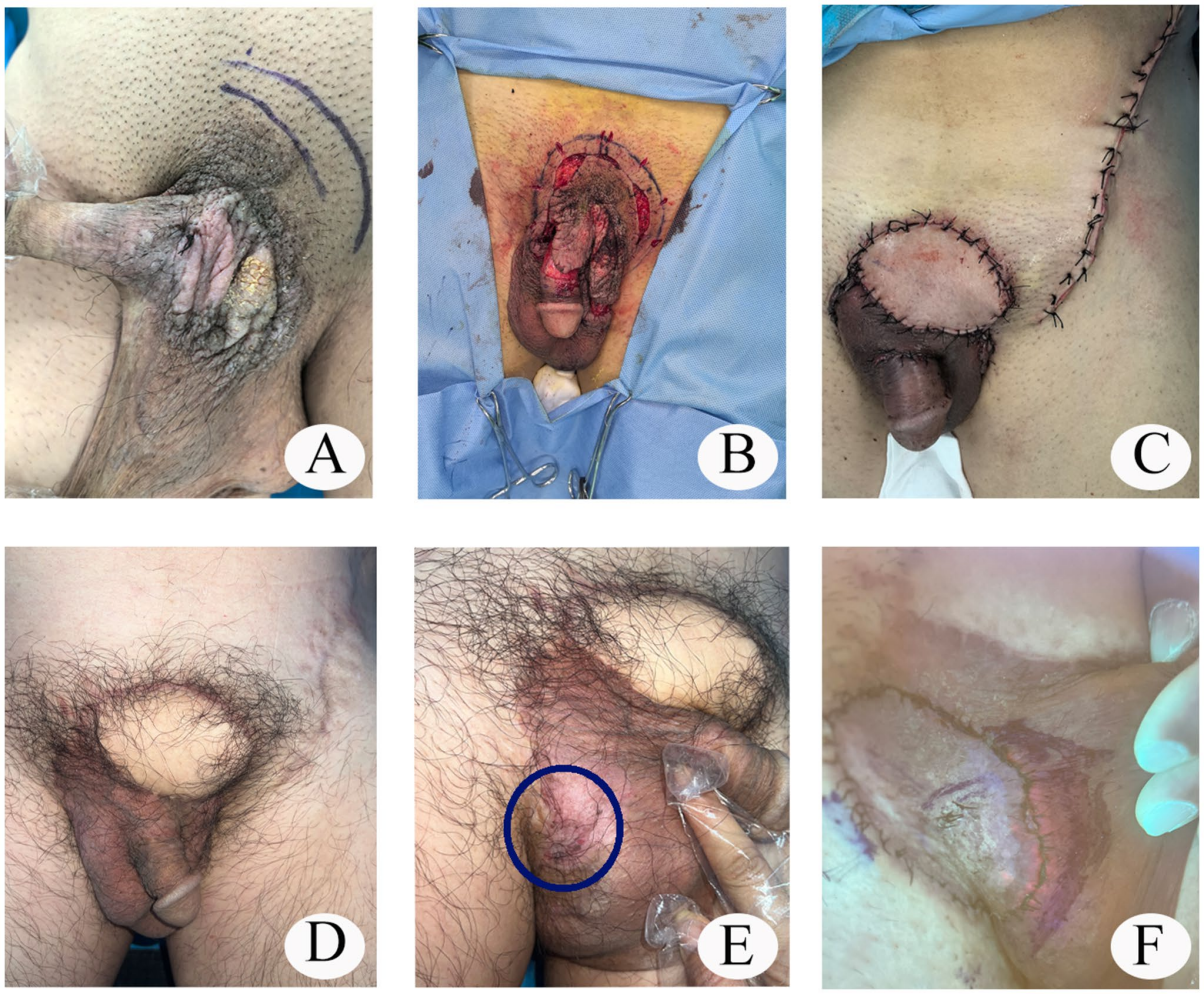
**(A)** Preoperative status of the lesion; **(B)** Mohs microscopic excision surgery; **(C)** A superficial circumflex iliac artery pedicled flap for wound restoration; **(D)** The condition 1 year post - surgery; **(E)** Recently diagnosed cutaneous lesion; **(F)** Determine the tumor boundary with the aid of photodynamic fluorescence technology.

The patient was a 53-year-old male. One year ago, he presented to our hospital due to erythema and thickening of the penis and scrotum, accompanied by pruritus (Figure 1A). The histopathological diagnosis was extramammary Paget’s disease. The first Mohs microscopic excision surgery was performed under general anesthesia (2 cm margin from the macroscopic tumor border) (Figure 1B), and the postoperative pathology showed residual tumor cells in the epidermis. Four days after the first surgery, a second wide resection (an additional 2 cm outward) was performed, and the postoperative pathology still showed tumor cells. Three days later, a third wide excision (an additional 2 cm outward) was performed under local anesthesia, and the postoperative pathology remained positive. Ten days later, a fourth wide excision (an additional 2 cm outward) was completed under general anesthesia, and the pathological margins were finally negative. Finally, the wound was covered by a superficial circumflex iliac artery pedicled flap and healed well (Figures 1C,D).

One year after the surgery, erythema recurred in the non-surgical area, and the histopathology confirmed EMPD (Figure 1E). Mohs microscopic excision surgery was performed under general anesthesia (an additional 2 cm outward), and the pathology showed tumor cells at the tissue margin. Before the second expansion resection, photodynamic fluorescence technology was used to assist in determining the tumor boundary: the lesion area was externally applied to 20% ALA, and was sealed in the dark for 3 h, then washed with normal saline, and observed under fluorescence to show a brick-red fluorescence in the tumor area, marking the boundary (Figure 1F), and then a wide excision (an additional 1.5 cm outward) was performed under general anesthesia, and the postoperative pathology showed a negative margin, and then a wound repair surgery was performed (6, 7). The patient has now been discharged and remains under regular follow-up.

## Discussion

EMPD is a rare type of tumor, commonly found in the external genitalia, accounting for 1–2% of all external genital tumors. It may originate from epidermal or skin appendage stem cells, and some studies suggest a possible origin from Toker cells (4, 8). Its pathological features include Paget cells visible within the epidermis, which can be distributed as single cells or in nests. Clinically, it often presents as multifocal, irregularly bordered skin lesions, making complete resection difficult, with a high recurrence rate (9, 10). Local extended resection is currently the main treatment method, but due to unclear boundaries, the accuracy of visual judgment of the resection margin is often inaccurate, with recurrence rate ranging from 20 to 60%. Although Mohs micrographic surgery (MMS) has been widely recommended, its recurrence rate remains at 12.2% (11, 12). Moreover, conventional MMS demonstrates significant limitations: the procedure is time-consuming, and multiple staged operations increase the burden on patients. Achieving an optimal balance between ensuring negative margins and preserving organ function remains particularly challenging when involving critical structures such as the urethra, anus, and clitoris (13). In the present case, the patient underwent four MMS procedures without achieving clear margins, which exemplifies the aforementioned clinical dilemma.

Photodynamic diagnosis works by the selective uptake of photosensitizers (such as ALA) by tumor cells, which are then metabolized into porphyrins and emit fluorescence under specific wavelengths of light, clearly delineating the tumor boundaries. It is particularly effective in revealing subclinical extension (8, 16), assisting in achieving more precise tumor resection while balancing tumor safety and tissue preservation. However, PDD has limitations in preoperative boundary assessment. False positives mainly result from inflammatory interference: inflammatory cells also take up ALA and produce fluorescence, causing false positive signals in non-tumor areas. Biopsy is a crucial step for eliminating false positives and improving diagnostic specificity. False negatives are more common in areas with sparse tumor cells or uneven drug penetration (such as the scrotum), which can lead to weak fluorescence signals or incomplete display (7). In this case, despite multiple Mohs surgeries and expanded resections, the margins remained positive, demonstrating that the tumor edges were unclear. Eventually, PDD was used to assist in determining the tumor boundaries and guide the surgical resection range. The postoperative routine pathology showed negative margins, effectively guiding the surgical range. It is noteworthy that this case presents a phenomenon contrary to conventional understanding: the tissue pathology shows a dense and strongly positive side of tumor cells, and after PDD calibration, the required expanded resection range was actually narrower; while the side with sparse and weakly positive tumor cells, the required expanded resection range was larger. This indicates that the functional tumor margin defined by PDD and the traditional pathological margin each have their own advantages and disadvantages. The two complement each other, helping to more comprehensively reflect the true biological boundary of tumors.

In recent years, apart from PDD, other novel fluorescence-guided techniques have also advanced. Su et al. (14) combined multichannel autofluorescence lifetime decay (MALD), fluorescence lifetime imaging microscopy (FLIM), and machine learning to assess margins at 12 sites within 10 min, featuring rapidity, objectivity, and a reduction in the burden on pathologists. Yoshida et al. (15) used fluorescent solvatochromic dyes in conjunction with two-photon microscopy to achieve high-resolution visualization of EMPD tumor boundary, clearly distinguishing tumor cells from surrounding keratinocytes. Although these techniques excel in precision and automation, PDD remains a promising clinical application prospect due to its advantages of simple equipment requirements, low cost, and ease of operation.

Surgical resection is the standard treatment for skin tumors, and achieving tumor-free surgical margins is crucial for reducing recurrence, especially for poorly demarcated tumors such as extramammary Paget’s disease (EMPD). Photodynamic technology offers a convenient and accurate means of identifying tumor boundaries, enabling the achievement of negative margins in a single procedure. This reduces the number of surgeries for the patient, improves surgical efficiency, and shortens hospital stays. Furthermore, it requires no complex preoperative preparation, minimizes patient discomfort, and lowers both physiological and economic burden on the patient.

We acknowledge several notable limitations of this study, including that it is a single case without a control group, a relatively short follow-up period, and the potential for interpretation bia in the assessment of fluorescence findings. Nevertheless, our preliminary results have demonstrated the advantages of PDD in defining the boundaries of refractory tumors, particularly its ability to reduce intraoperative trauma and surgical risk while offering a novel strategy for precision resection that balances oncologic safety with functional preservation.

In conclusion, photodynamic diagnosis technology has significant advantages in locating the boundaries of EMPD, and is expected to improve surgical success rates and patient prognosis.

## Data Availability

The original contributions presented in the study are included in the article/supplementary material, further inquiries can be directed to the corresponding author.
